# Supplementary material for: Recurrent viral capture of cellular phosphodiesterases that antagonize OAS-RNase L
Source: Proc Natl Acad Sci U S A. 2024 Jan 26;121(5):e2312691121. doi: 10.1073/pnas.2312691121 (PMC10835031; doi:10.1073/pnas.2312691121)
Supplement: Supplementary file 1 — Appendix 01 (ZIP) [file pnas.2312159120.sapp.zip › Supplementary Files/Supplementary File 4.pdf]

Sequencing Run	Mean Read Length (bases)	Median Read Length (bases)	# Total Reads	Total bases	MHV-aligned bases	% MHV-aligned bases	Mean MHV Cover	Mean Coverage ORF1ab	Mean Coverage AKAP7	Mean Relative Coverage AKAP7	Mean Coverage ORF2	Mean Relative Coverage ORF2
passage 0	1,111.60	661	65,596.00	72,917,026.00	54,933,003.00	75.33631857	3892.691385x	217.952476x	2442.20712x	11.20522769	247.3435115x	1.134850661
macrophage plaque 1	1,343.80	1,190.00	382,284.00	513,721,354.00	360,557,983.00	70.18551598	11296.08303x	319.6635262x	8882.134304x	27.78588602	596.9631043x	1.867473313
L2 plaque 1	1,315.20	1,022.00	88,349.00	116,199,691.00	83,921,621.00	72.22189687	2603.023655x	368.8556644x	35.80582524x	0.097072727	591.8473282x	1.604549924
L2 plaque 2	1,223.30	743	528,118.00	646,044,956.00	463,744,461.00	71.78207286	13970.76232x	2236.325335x	242.4530744x	0.108415833	3271.125954x	1.462723649
L2 plaque 3	1,065.20	836	336,606.00	358,556,880.00	262,272,278.00	73.14663102	8177.027823x	595.5423029x	82.79449838x	0.139023707	1117.840967x	1.877013541
L2 plaque 4	1,110.30	808	1,045,172.00	1,160,468,881.00	875,067,357.00	75.40636128	27296.16375x	2930.50847x	3263.919094x	1.113772278	3696.030534x	1.261224996
L2 plaque 5	1,528.00	1,103.00	199,537.00	304,889,621.00	212,104,890.00	69.56776334	6666.61502x	567.4103588x	113.1682848x	0.199446984	1212.394402x	2.136715313
