# Supplementary material for: Recurrent viral capture of cellular phosphodiesterases that antagonize OAS-RNase L
Source: Proc Natl Acad Sci U S A. 2024 Jan 26;121(5):e2312691121. doi: 10.1073/pnas.2312691121 (PMC10835031; doi:10.1073/pnas.2312691121)
Supplement: Supplementary file 1 — Appendix 01 (ZIP) [file pnas.2312159120.sapp.zip › Supplementary Files/Supplementary File 5.rtf]

=== All Lineages (51233 total) ===<10: 95.95573165733023%10-100: 2.88290750102473%>100: 1.1613608416450334%=== Lineage B.1.1.7 (45290 total) ===<10: 97.57120777213512%10-100: 1.9209538529476706%>100: 0.5078383749172003%=== Lineage XBB.1 (627 total) ===<10: 88.03827751196172%10-100: 6.0606060606060606%>100: 5.901116427432217%=== Lineage XBB.1.16 (518 total) ===<10: 86.48648648648648%10-100: 12.162162162162161%>100: 1.3513513513513513%=== Lineage XBB.1.5 (4373 total) ===<10: 81.86599588383261%10-100: 11.022181568717128%>100: 7.111822547450263%=== Lineage XBB.1.9.1 (425 total) ===<10: 92.0%10-100: 5.647058823529412%>100: 2.3529411764705883%
